# EndoPredict^®^ in early hormone receptor-positive, HER2-negative breast cancer

**DOI:** 10.1007/s10549-020-05688-1

**Published:** 2020-05-20

**Authors:** K. Almstedt, S. Mendoza, M. Otto, M. J. Battista, J. Steetskamp, A. S. Heimes, S. Krajnak, A. Poplawski, A. Gerhold-Ay, A. Hasenburg, C. Denkert, M. Schmidt

**Affiliations:** 1 Department of Obstetrics and Gynecology, University Medical Center Mainz, Langenbeckstr. 1, 55131 Mainz, Germany; 2 Institute for Molecular Pathology, Trier, Germany; 3 Institute of Medical Biometry, Epidemiology and Informatics (IMBEI), University Medical Center Mainz, Mainz, Germany; 4 Institute of Pathology, Philipps-University Marburg and UKGM Marburg, Marburg, Germany

**Keywords:** Early breast cancer, EndoPredict^®^, Endocrine therapy, Treatment decision, Prognosis, Gene expression

## Abstract

**Purpose:**

Evaluating consecutive early breast cancer patients, we analyzed both the impact of EndoPredict^®^ on clinical decisions as well as clinico-pathological factors influencing the decision to perform this gene expression test.

**Methods:**

Hormone receptor (HR)-positive and human epidermal growth factor receptor 2 (HER2)-negative early breast cancer patients treated between 2011 and 2016 were included in this study to investigate the role of EndoPredict^®^ (EPclin) in the treatment of early breast cancer. A main study aim was to analyze the changes in therapy recommendations with and without EPclin. In addition, the impact of clinico-pathological parameters for the decision to perform EPclin was examined by Pearson's chi-squared test (*χ*^2^-test) and Fisher's exact test as well as univariate and multivariate logistic regressions.

**Results:**

In a cohort of 869 consecutive early HR-positive, HER-negative breast cancer patients, EPclin was utilized in 156 (18.0%) patients. EPclin led to changes in therapy recommendations in 33.3% (*n* = 52), with both therapy escalation in 19.2% (*n* = 30) and de-escalation in 14.1% (*n* = 22). The clinico-pathological factors influencing the use of EPclin were age (*P* < 0.001, odds ratio [OR] 0.498), tumor size (*P* = 0.011, OR 0.071), nodal status (*P* = 0.021, OR 1.674), histological grade (*P* = 0.043, OR 0.432), and Ki-67 (*P* < 0.001, OR 3.599).

**Conclusions:**

EPclin led to a change in therapy recommendations in one third of the patients. Clinico-pathological parameters such as younger age, smaller tumor size, positive nodal status, intermediate histological grade and intermediate Ki-67 had a significant influence on the use of EndoPredict^®^.

**Electronic supplementary material:**

The online version of this article (10.1007/s10549-020-05688-1) contains supplementary material, which is available to authorized users.

## Introduction

The extent of the benefit from adjuvant chemotherapy depends on the patient’s individual risk profile [1]. According to the current recommendations, traditional clinico-pathological parameters such as tumor size, nodal status, histological grading, hormone receptor (HR) status and human epidermal growth factor receptor 2 (HER2) status should be used for risk stratification in women with early stage invasive breast cancer [2, 3]. In clinical practice, breast cancer is subdivided into different subtypes (i.e., luminal A-like, luminal B-like, HER2-like, triple-negative) [3]. The development and use of genomic tests using gene expression profiling has the aim to improve prediction of clinical outcome. Especially for HR-positive, HER2-negative, node-negative tumors, the additional use of multigene assays should help to reliably assess the risk profile in order to avoid both under- and overtreatment.

For clinical use, it is crucial that gene expression analyses are assessed according to clear evidence-based criteria [4]. Recommended commercially available biomarker assays by the St. Gallen International Consensus Conference and the American Society of Clinical Oncology (ASCO) are Oncotype DX^®^, EndoPredict^®^, MammaPrint^®^, PAM50/Prosigna^®^ and Breast Cancer Index [2, 3, 5, 6]. All of these gene expression assays are endorsed for guiding the decision on adjuvant chemotherapy in hormone receptor-positive, node-negative tumors if all other criteria do not allow a therapeutic decision. MammaPrint^®^, EndoPredict^®^ and Prosigna^®^ might also be used in 1–3 involved axillary lymph nodes. Retrospective evidence exists for the use of OncotypeDX^®^ in node positive disease [7, 8], although the prospective phase 3 RxSPONDER trial (SWOG S1007, https://clinicaltrials.gov/ct2/show/NCT01272037) is still ongoing. For the 21-gene recurrence score (TAILORx trial) [9–11] and the 70-gene signature (MINDACT trial) [12] prospective data are available. There is also a smaller prospective study on EndoPredict^®^ in 200 patients, which addresses the clinical and psychological impact of this multigene assay [13]. In addition, there is ample prospective-retrospective evidence for the approved multigene assays.

The RNA-based multigene score EndoPredict^®^ (EP, Myriad Genetics^®^, Salt Lake City, USA) is based on the gene expression analysis of 12 genes, eight cancer-related, three reference genes and one control gene for DNA contamination. EndoPredict^®^ integrates genomic and clinical information by including clinico-pathological parameters like tumor size and number of involved lymph nodes (EPclin). Integrating these two clinical parameters to the molecular test (EPclin) increased the prognostic value especially for late-distant recurrence for the multigene assay EndoPredict^®^ [14]. The test result distinguishes EPclin low-risk and EPclin high-risk patients [15–17]. The clinical benefit of EndoPredict^®^ to identify a subgroup with an excellent prognosis with endocrine therapy only is well-established [15, 18–20].

With an increasing use of multigene tests, it is of utmost importance to know whether their use changes treatment decisions. This retrospective study in consecutive patients compared pre- and post-test therapy decisions and changes in treatment recommendations. Furthermore, we evaluated clinico-pathological factors influencing the decision for the additional use of EPclin. These real-world data should be of special interest for indication and evaluation of EndoPredict^®^.

## Methods

### Patient cohorts and data collection

**Fig. 1:**
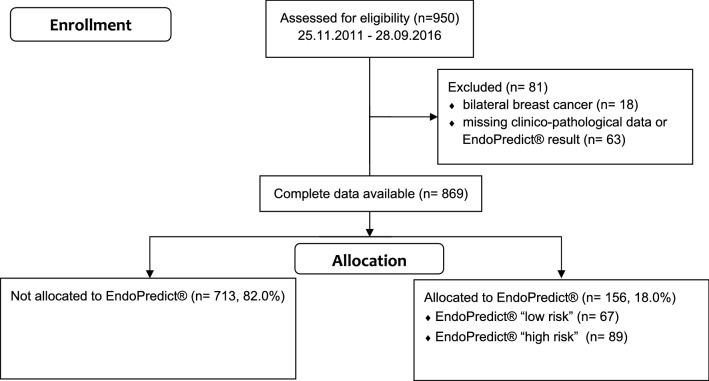
Patient enrollment

The certified breast cancer center of the University Medical Center Mainz has been prospectively documenting clinico-pathological characteristics as well as therapies of all treated breast cancer patients. This database was searched for patients with hormone receptor-positive, HER2-negative early breast cancer, with a maximum of three positive axillary lymph nodes diagnosed between November 2011 and September 2016. Patients with neoadjuvant chemotherapy, distant metastasis or breast cancer recurrence were not eligible for this study (Fig. 1).

Information on patient characteristics (e.g., age at primary breast surgery, menopausal status), histopathological data (tumor size, axillary lymph-node status, histological tumor type, histological grading, estrogen receptor (ER)-, progesterone receptor (PR)-, and HER2-status, proliferation index assessed by Ki-67), therapeutic recommendations as well as the decision to use EndoPredict^®^, were collected from the patients’ medical records and the documented tumor board decisions. The results of the EndoPredict^®^ test (EP- and EPclin test) were also obtained from this source.

### Gene expression analysis (EndoPredict^®^)

EndoPredict^®^ was performed either in the Institute of Pathology of the Charité, University Medical Center Berlin, Campus Mitte, Charitéplatz 1, 10117 Berlin (*n* = 8) or in the Molecular Pathology Trier, Max-Planck-Str. 17, 54296 Trier (*n* = 148) using RNA from formalin-fixed and paraffin-embedded (FFPE) tissue from the surgical specimen. EP was combined with tumor size and nodal status into a comprehensive risk score, EPclin, as previously described [15]. The EPclin-score ranged from 0 to 7. EPclin-score ≥ 3.3 differentiated between high-risk and low-risk patients.

To assess a possible change in the treatment decision, the recommendation of the tumor conference with knowledge of the EPclin result (post-test) was compared with the original recommendation (pre-test).

### Statistical analysis

The primary objective was to assess differences in pre- and post-test therapy decisions. The secondary objectives of this retrospective study were (i) to evaluate clinico-pathological factors influencing the decision for the application of EPclin in HR-positive, HER2-negative early breast cancer patients with 0–3 involved axillary lymph nodes as well as the relationship between EndoPredict^®^ and (ii) clinico-pathological variables, and (iii) molecular subtypes. Patient characteristics were presented in absolute and relative numbers. Comparisons between clinico-pathological factors and the results of the EndoPredict^®^ were calculated using the Pearson's chi-squared test (*χ*^2^-test) and Fisher’s exact test. Afterwards, univariate logistic regressions were performed including one of the following independent variables: age at diagnosis, menopausal status (pre-/perimenopausal vs. postmenopausal), breast tumor size (pT1 vs. pT2 vs. pT3/pT4), axillary nodal status (pN0 vs. pN1), histological grade of differentiation (grade I vs. II vs. III), Ki-67 (< 20% vs. 20–40% vs. > 40%), ER status (positive vs. negative), PR status (positive vs. negative), and cancer molecular subtype (luminal A-like/Ki-67 < 20% vs. luminal B-like/Ki-67 ≥ 20%). All independent variables were included for the multivariable analysis and the best fitting model was obtained utilizing backward selection by AIC. All associations were reported as odds ratios (OR) including their 95% confidence interval (CI) and *P* values. All *P* values are two-sided, and *P* < 0.05 was considered significant. As all analyses were explorative and not adjusted for multiple testing, the *P* values should be interpreted with caution and in connection with the effect estimates. Statistical analyses were performed using R (v. 3.6.2) [R Development Core Team (2008). R: A language and environment for statistical computing. R Foundation for Statistical Computing, Vienna, Austria. ISBN 3-900051-07-0, https://www.R-project.org.]. The study was approved by the Research Ethics Committee of the University Medical Center Mainz (Mainz, Germany). Informed consent was obtained from all patients.

## Results

### Patient population

**Fig. 2:**
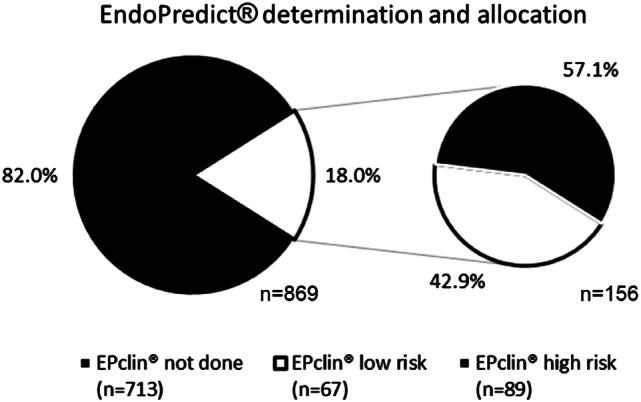
EndoPredict^®^ determination and allocation

950 patients with hormone receptor-positive, HER2-negative early breast cancer, with a maximum of three positive axillary lymph nodes, were screened. 869 (91.5%) patients were eligible for evaluation. Patients were excluded due to bilateral breast cancer (*n* = 18) or missing clinico-pathological data or missing EndoPredict^®^ result (*n* = 63) (Fig. 1). In 156 (18.0%) patients, EndoPredict^®^ was performed based on the clinician's decision. EndoPredict^®^ test results classified 67 patients (42.9%) as low-risk and 89 patients (57.1%) as high-risk (Fig. 2). In the entire cohort, adjuvant chemotherapy was recommended for 271 patients (31.2%).

### Impact of EndoPredict^®^ for adjuvant treatment decisions

**Fig. 3:**
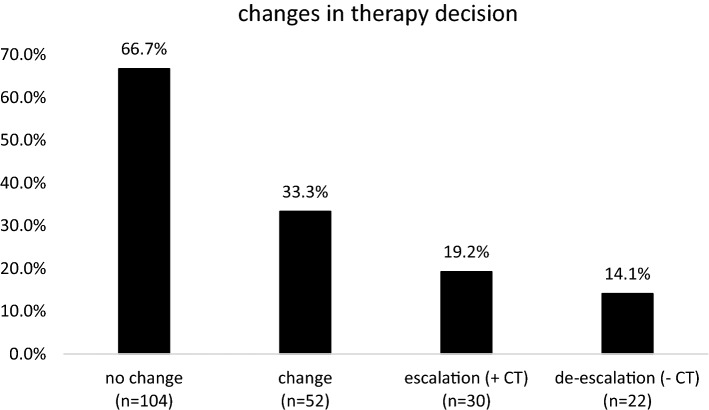
Changes in therapy decision

According to the EPclin results, chemotherapy was recommended in 57.1% (*n* = 89) whilst no chemotherapy was advised in 42.9% (*n* = 67) (post-test). Without knowledge of EPclin, chemotherapy was recommended in 81 patients (51.9%) and not recommended in 75 patients (48.1%) (pre-test) (Appendix 1, Only Online). In detail, 104 patients (66.7%) received the identical treatment recommendation pre- and post-test. In 33.3% (*n* = 52), however, there was an adjustment of the pre-test therapy decision after knowledge of EPclin became available. In 30 patients (19.2%), adjuvant chemotherapy was recommended post-test compared to the pre-test “low risk” classification based on clinical-pathological parameters (*n* = 75). In 45 patients there was no change to an endocrine therapy recommendation. Conversely, in 22 patients (14.1%) adjuvant chemotherapy was omitted (EPclin “low risk”). Based on clinico-pathological classification, adjuvant chemotherapy was advised in 81 patients, i.e., adjuvant chemotherapy was furthermore recommended in 59 patients (Appendix 1, Only Online) (Fig. 3).

Among patients who had not received an EndoPredict^®^ test (*n* = 713), chemotherapy was recommended to 182 patients (25.5%). For the remaining 531 patients (74.5%) chemotherapy was not advised.

### Influence of clinico-pathological factors on the application of EndoPredict^®^

The traditional clinico-pathological prognostic factors were examined in univariate analysis with respect to the decision to perform EPclin (Table 1). Multivariate analysis included all parameters that showed a significant result in the univariate testing: age at primary breast surgery (*P* < 0.001), tumor size (*P* = 0.014), nodal status (*P* < 0.001), histological grade (*P* < 0.001), Ki-67 (*P* < 0.001) and molecular subtype (*P* < 0.001) [21]. Younger age (OR 0.498, 95% CI 0.335–0.734, *P* < 0.001), smaller tumor size (OR 0.071, 95% CI 0.004–0.354, *P* = 0.011), positive nodal status (OR 1.674, 95% CI 1.080–2.587, *P* = 0.021), low/intermediate histological grade (OR 0.432, 95% CI 0.192–0.984, *P* = 0.043), and intermediate Ki-67 (OR 3.599, 95% CI 2.370–5.508, *P* < 0.001) retained an higher chance for EPclin (Table 2).

**Table 1:** Associations between EndoPredict^®^ and clinico-pathological characteristics (*n* = 869)

	Total number of patients (*n* = 869)	EP test		*P* value
		Not done (*n* = 713)	Done (*n* = 156)	
Age at primary breast surgery				**< 0.001**
Mean (years)	61	62	57	
Minimum (years)	28	28	32	
Maximum (years)	106	106	75	
Menopausal status				0.634
Pre-/perimenpausal	244 (28.1%)	198 (27.8%)	46 (29.5%)	
Postmenopausal	625 (71.9%)	515 (72.2%)	110 (70.5%)	
Histopathologic classification				0.394
Invasive carcinoma of no special type (NST)	678 (78.0%)	557 (78.1%)	121 (77.6%)	
Invasive lobular carcinoma	120 (13.8%)	96 (13.5%)	24 (15.4%)	
Others	71 (8.2%)	60 (8.4%)	11 (7.1%)	
Tumor size				**0.014**
pT1	558 (65.2%)	466 (65.4%)	92 (59.0%)	
pT2	276 (31.8%)	213 (29.9%)	63 (40.4%)	
pT3/pT4	35 (4.0%)	34 (4.8%)	1 (0.6%)	
Nodal status				**< 0.001**
pN0	686 (78.9%)	582 (81.6%)	104 (66.7%)	
pN1	183 (21.1%)	131 (18.4%)	52 (33.3%)	
Histological grade				**< 0.001**
G1	244 (28.1%)	227 (31.8%)	17 (10.9%)	
G2	466 (53.6%)	353 (49.5%)	113 (72.4%)	
G3	159 (18.3%)	133 (18.7%)	26 (16.7%)	
Estrogen receptor status				0.934
Positive	863 (99.3%)	708 (99.3%)	155 (99.4%)	
Negative	6 (0.7%)	5 (0.7%)	1 (0.6%)	
Progesterone receptor status				0.825
Positive	784 (90.2%)	644 (90.3%)	140 (89.7%)	
Negative	85 (9.8%)	69 (9.7%)	16 (10.3%)	
Ki-67				**< 0.001**
Low (< 20%)	547 (62.9%)	484 (67.9%)	63 (40.4%)	
Intermediate (20–40%)	268 (30.8%)	180 (25.2%)	88 (56.4%)	
High (> 40%)	54 (6.2%)	49 (6.9%)	5 (3.2%)	
Molecular subtype				**< 0.001**
Luminal A-like	547 (62.9%)	484 (67.9%)	63 (40.4%)	
Luminal B-like	322 (37.1%)	229 (32.1%)	93 (59.6%)	
EndoPredict^®^ risk category				
Low (≤ 3.3)	67	0	67 (42.9%)	
High (> 3.3)	89	0	89 (57.1%)	

Bold indicates *P* < 0.05

**Table 2:** Influence of clinico-pathological factors on the use of EndoPredict^®^ (multivariate analysis)

	*P* value	Odds ratio	95% confidence interval	
Age at primary breast surgery				
≤ 60 years	Ref			
> 60 years	**< 0.001**	0.498	0.335	0.734
Tumor size				
pT1	Ref			
pT2	0.343	0.824	0.550	1.226
pT3/4	**0.011**	0.071	0.004	0.354
Nodal status				
Negative	Ref			
Positive	**0.021**	1.674	1.080	2.587
Histological grade				
G1	Ref			
G2	0.806	1.086	0.572	2.153
G3	**0.043**	0.432	0.192	0.984
Ki-67				
Low (≤ 20%)	Ref			
Intermediate (20–40%)	**< 0.001**	3.599	2.370	5.508
High (≤ 40%)	0.911	1.061	0.335	2.818

Bold indicates *P* < 0.05

### Relationship between EndoPredict^®^ and clinico-pathological variables

The common clinico-pathological prognostic factors were examined in all patients with available EPclin (*n* = 156) using univariate logistic regression with respect to the EndoPredict^®^ result. Only pT2 tumor size (*P* = 0.007) and positive nodal status (*P* = 0.031) showed a higher chance for EPclin risk category (Table 3). The sample size for pT3/4 tumor size (*n* = 1) and for higher Ki-67 (*n* = 5) were too small for stable estimation.

**Table 3:** Association between EndoPredict^®^ result and patient and tumor characteristics (*n* = 156)

	EndoPredict^®^ done (*n* = 156)	EndoPredict^®^ result		*P* value [OR]
		Low risk (*n* = 67)	High risk (*n* = 89)	
Age at primary breast surgery				
Mean (years)	57	70	56	
Minimum (years)	32	68	35	
Maximum (years)	75	72	75	
≤ 60 years	90 (57.7%)	37 (55.2%)	53 (59.6%)	Ref
> 60 years	66 (42.3%)	30 (44.8%)	36 (40.4%)	0.588 [0.838]
Menopausal status				
Pre-/perimenpausal	46 (29.5%)	17 (25.4%)	29 (32.6%)	Ref
Postmenopausal	110 (70.5%)	50 (74.6%)	60 (67.4%)	0.329 [0.703]
Histopathologic classification				
Invasive carcinoma of no special type (NST)	121 (77.6%)	53 (79.1%)	68 (76.4%)	Ref
Invasive lobular carcinoma	24 (15.4%)	11 (16.4%)	13 (14.6%)	0.855 [0.921]
Others	11 (7.1%)	3 (4.5%)	8 (9.0%)	0.297 [2.078]
Tumor size				
pT1	92 (59.0%)	48 (71.6%)	44 (49.3%)	Ref
pT2	63 (40.4%)	19 (28.4%)	44 (49.3%)	**0.007 [2.526]**
pT3/pT4	1 (0.6%)	0	1 (1.1%)	
Nodal status				
pN0	104 (66.7%)	51 (76.1%)	53 (59.6%)	Ref
pN1	52 (33.3%)	16 (23.9%)	36 (40.4%)	**0.031 [2.165]**
Histological grade				
G1	17 (10.9%)	9 (13.4%)	8 (9.0%)	Ref
G2	113 (72.4%)	47 (70.1%)	66 (74.2%)	0.381 [1.580]
G3	26 (16.7%)	11 (16.4%)	15 (16.9%)	0.495 [1.534]
Estrogen receptor status				
Positive	155 (99.4%)	67 (100%)	88 (98.9%)	Ref
Negative	1 (0.6%)	0	1 (1.1%)	0.987
Progesterone receptor status				
Positive	140 (89.7%)	63 (94.0%)	77 (86.5%)	Ref
Negative	16 (10.3%)	4 (6.0%)	12 (13.5%)	0.136 [2.454]
Ki-67				
Low (< 20%)	63 (40.4%)	33 (49.3%)	30 (33.7%)	Ref
Intermediate (20–40%)	88 (56.4%)	33 (49.3%)	55 (62.8%)	0.070 [1.833]
Higher (> 40%)	5 (3.2%)	1 (1.5%)	4 (4.5%)	
Molecular subtype				
Luminal A-like	63 (40.4%)	33 (49.3%)	30 (33.7%)	Ref
Luminal B-like	93 (59.6%)	34 (50.7%)	59 (66.3%)	0.051 [1.909]

Bold indicates *P* < 0.05

### Relationship between EndoPredict^®^ and molecular subtypes

EndoPredict^®^ classified 33 Luminal A-like tumors (49.3% of all low-risk tumors) as low risk and 30 (33.7% of all high-risk tumors) as high risk. In Luminal B-like tumors, EPclin allocated 34 cases (50.7%) in the low risk and 59 cases (66.3%) in the high risk category, respectively (*P* = 0.051) (Table 3).

## Discussion

In our analysis of 869 consecutive HR-positive, HER2-negative breast cancer patients, gene expression analysis with EPclin was performed in 18.0% for adjuvant treatment decisions. In 33.3% use of EPclin led to a change in treatment recommendations.

Using EPclin, 67 (42.9%) patients were assigned low-risk and 89 (57.1%) high-risk. Remarkably, the distribution between the low and high risk category was different from other studies. In contrast to our results, more low-risk patients were observed in the cohort used for EPclin validation. In this study, 63% of 1702 patients were classified as low-risk and 37% as high-risk [18, 22]. Similarly, the low-risk group of postmenopausal patients from the TransATAC study (58.8%) was also considerably larger than in the present study [14]. However, both studies included patients of a prospective study population for whom mainly endocrine therapy was indicated. Therefore the ABCSG-6 /-8 and the TransATAC study cohort represented a completely different study population than the current real-world population in our study. Most likely, a clinical pre-selection to favorable risk might explain this difference. Conversely, another study investigating two different chemotherapy regimens in early breast cancer assigned only 25% to the low-risk group [16]. Nonetheless, a risk distribution comparable to our results was shown by Müller et al., who evaluated all EndoPredict^®^ requests in a single pathology institute during one year. 46.4% were classified as low-risk and 53.5% as high-risk [23]. Only a minority of the patients in our study (*n* = 8) was also included in the aforementioned analysis. However, in contrast to their analysis, our study included all eligible HR-positive, HER2-negative patients with 0–3 involved axillary lymph nodes over a period of five years, providing a more unbiased view on the use of EPclin in early breast cancer. The indication to perform a gene expression assay may also be influenced by the experience of the physicians ordering the test, in particular by their individual threshold for administration of chemotherapy. This selection bias could explain the different risk-profiles in different EndoPredict^®^ cohorts.

In our analysis, the decision to perform EPclin was multivariate associated with younger age, smaller tumor size, positive nodal status, low/intermediate histological grade and intermediate Ki-67 in multivariate testing. The younger median age in the EPclin-tested cohort (57 years) was similar to the observations in other studies [23–25]. The younger age in breast cancer patients is an unfavorable prognostic factor. More aggressive tumor therapy, however, is associated with the increased probability of long-term side effects. Therefore, a reason for the additional performance of a multigene assay in younger patients is primarily whether chemotherapy can be safely avoided. Conversely if a luminal-like tumor is present in elderly patients, endocrine therapy alone is more likely. Histological grade and the proliferative marker Ki-67 are important variables for the risk classification of early breast cancer. Both variables, however, have considerable inter-laboratory and -observer variability [26, 27]. Therefore, it is not surprising that, similar to other reports, the additional gene expression analysis is most often used in tumors of intermediate histological grade [23] and intermediate Ki-67. Median Ki-67 was 20% in the EP cohort v. 10% in the untested patient group. Accordingly, in a large study by Nitz and co-workers, patients with intermediate Ki-67 (> 10% to < 40%) were most likely to benefit from a gene expression test [28]. Histological grade as an influencing factor for use of multigene assays has also been observed in other settings with an increased percentage of G2 tumors in cohorts tested with Oncotype DX^®^ [25, 29]. In the assessment of the relationship between EndoPredict^®^ and clinico-pathological variables only T2 tumor size (*P* = 0.007) and positive nodal status (*P* = 0.031) showed a higher chance for EPclin risk category (Table 3). Among the multigene tests, the EPclin test is the only one that integrates both clinical parameters (tumor size and nodal status) in addition to molecular genetic parameters. Thus, our results underline the strong influence of these two clinical factors on the patient’s individual risk profile with breast cancer.

We have shown that in 33.3% EPclin led to changes in the therapy recommendation. Comparable rates of therapy changes have been observed in other studies. Penault-Llorca et al. showed in a prospective study that EPclin resulted in a change of therapy recommendation in one third of patients [13]. Similarly, Müller et al. reported a change in 37.7% [23]. In our study, EPclin led in 19.2% to therapy escalation and in 14.1% to de-escalation. Interestingly, both studies described therapy de-escalation considerably more often than escalation, presumably reflecting both differences in the patient population as well as in the willingness to recommend adjuvant chemotherapy in intermediate-risk HR-positive, HER2-negative early breast cancer. In line with the latter argument is the fact that in our patients without gene expression profiling the rate of chemotherapy recommendation was only 25.5%. Differences of 30–50% between pre- and post-test treatment recommendations were also shown in studies using Oncotype DX^®^ and the 70-gene signature (MammaPrint^®^) [12, 30–32].

Our study has some strengths and limitations. A potential weakness is that our study was retrospective and performed in a single certified breast cancer center. Furthermore, patient preferences were not addressed. An extension of our study endpoints to patient-related questions (e.g., expectations and wishes on a gene signature test, impact of the test result for the patient), could have led to valuable additional information. However, a major strength of our study is the consecutive inclusion of patients which enables the assessment of EPclin in the context of well-established clinic-pathological factors. To the best of our knowledge, this is the first study dealing with this topic using real-world data. In our study post-test results of EndoPredict^®^ were the reason for a change in clinical decisions in more than 1/3 of tested patients. This is a valuable finding in a "real life" use of this multigene test. With an increasing use of multigene assays it is of particular interest for the indication and evaluation of the EPclin results to know if the clinical treatment decision changes.

In conclusion, EPclin was only performed in a subset of consecutive HR-positive, HER2-negative early breast cancer patients. However, EPclin resulted in a considerable change in therapy recommendations in one third of patients, potentially reducing over- and under-treatment in early breast cancer patients.

## Electronic supplementary material

Below is the link to the electronic supplementary material.

Supplementary file1 (DOCX 13 kb)
