# Supplementary material for: EndoPredict® in early hormone receptor-positive, HER2-negative breast cancer
Source: Breast Cancer Res Treat. 2020 May 20;182(1):137–46. doi: 10.1007/s10549-020-05688-1 (PMC7275019; doi:10.1007/s10549-020-05688-1)
Supplement: Supplementary file 1 — Supplementary file1 (DOCX 13 kb) [file 10549_2020_5688_MOESM1_ESM.docx]

Appendix 1: Recommendations for adjuvant chemotherapy (EndoPredict® vs clinico-pathological risk categories, n=156; p<0.001)

|  |  | **Clinic-pathological risk categories** | |  |
| --- | --- | --- | --- | --- |
| **EndoPredict®** |  | **Recommended (“high risk”)** | **not recommended (“low risk”)** | **all** |
|  | **Recommended**  **(EPclin “high risk”)** | 59^+^ | 30^*^ | 89  (57.1%) |
|  | **Not**  **recommended**  **(EPclin “low risk”)** | 22^*^ | 45^+^ | 67  (42.9%) |
|  | **all** | 81  (51.9%) | 75  (48.1%) | 156  (100.0%) |

^+^ patients with the same treatment recommendation regardless of EndoPredict® result (n=104, 66.7%)

* patients with a discordance between the therapy decision with or without knowledge of EndoPredict® (n= 52, 33.3%)
